# Standard feeding strategies with natural insemination improved fertility in repeat breeding dairy cows

**DOI:** 10.5455/javar.2021.h513

**Published:** 2021-06-23

**Authors:** Mir Md. Iqbal Hasan, Moinul Hasan, Mohammad Harun-Or-Rashid, Marzia Rahman, Md. Siddiqur Rahman, Nasrin Sultana Juyena

**Affiliations:** 1 Department of Surgery and Obstetrics, Bangladesh Agricultural University, Mymensingh, Bangladesh; 2 Department of Physiology, Sylhet Agricultural University, Sylhet, Bangladesh; 3 Department of Microbiology and Hygiene, Bangladesh Agricultural University, Mymensingh, Bangladesh.; 4 Department of Medicine, Bangladesh Agricultural University, Mymensingh, Bangladesh

**Keywords:** Artificial insemination, natural insemination, hemato-biochemical parameters, pregnancy rate, standard feeding, traditional feeding

## Abstract

**Objective:**

The experiment was designed to establish suitable management strategies through the different feeding and breeding approaches on fertility improvement in the experimental repeat breeding (RB) cows.

**Materials and Methods:**

80 RB cows were selected for this experiment. Before grouping, all cows were deworming and then divided into four equal groups, namely Group–TF1 [traditional feeding practice and natural insemination (NI)], Group–TF2 [traditional feeding practice and Artificial insemination (AI)], Group–SF1 [standard (STD) feeding practice and NI], and Group–SF2 (STD feeding practice and AI). These allocated RB cows were fed by traditional and STD feeding methods for 90 days and then inseminated by AI and NI breeding systems. The dominant follicle (DF) diameter, hemato-biochemical elements, and estrogen (E_2_) hormone were estimated during the insemination of cows. Estimation of the pregnancy rate was carried out at days 45–90 post-insemination in the cows.

**Results:**

The pregnancy rate was significantly (*p *< 0.05) higher in STD feeding practice with NI when compared to traditional feeding practice irrespective of breeding systems, and it was also significantly (*p *< 0.05) higher in NI than in AI breeding system, irrespective of feeding strategies. The results also showed that the diameter of DF, serum E_2_, total erythrocyte count, hemoglobin, packed cell volume, total cholesterol, total protein, glucose, calcium, phosphorus, ferric iron, copper, zinc, and magnesium at the time of insemination were significantly (*p *< 0.01) elevated in the experimental RB cows with STD feeding practice. The diameter of DF and serum E_2_ were significant (*p *< 0.01) and positively correlated with all hemato-biochemical elements in the cows at the time of insemination.

**Conclusion:**

The results suggest that NI with STD feeding practice may increase fertility in RB cows by improving general health status. Finally, it could support the veterinarians and researchers to define the management strategies using feeding and breeding strategies to prevent repeat breeding syndrome in dairy cows.

## Introduction

In crossbred cows, the most critical indicators for reproductive disorders are excessive milk production and imbalanced dieting [1]. Poor fertility in the cows is due to improper supply of various nutrients, minerals, vitamins, and trace elements [2]. Moreover, poor metabolic and nutritional conditions negatively relate to dairy cows’ reproduction, although mortality is not usual [3]. Early lactation of a highly milking cow has negative energy balance and disturbances of endocrine and biochemical synchrony with an ovarian follicular wave, which affect the oocyte quality, like growing and maturing of the oocyte [2,3]. Decreases in certain biochemicals and antioxidant molecules in the body fluids around birth have been associated with immune dysfunction due to a higher chance of endometrial diseases that threaten fertility. Furthermore, disrupted metabolic health status enhances immunosuppression which also lessens reproduction [3].

The standard (STD) feeding practice of domestic animals is not well established, and it is very poor in Bangladesh [4]. The owners have no scientific knowledge regarding the STD feeding of dairy cows to fulfill the essential nutrients. In the traditional feeding strategies for dairy herd management, stall-feeding and seldomly tethered on roadsides and fallow land are practiced due to unavailable grazing lands in Bangladesh. Moreover, the periodic and improper supply of hay and raw grass leads to a higher obstacle in feeding dairy cows [5]. Dairy farm owners commonly use the best feeding through supplementation of concentrate byproducts in milking cows, as they get instant yields of milk [6], and this strategy is very common in the smallholding dairy farms in Bangladesh.

On the contrary, research proposes that feeding in postpartum cows should be proper and sufficient to maintain the body, milk yield, and to induct the ovarian rhythm [7]. Enough nutrition is necessary during the pre-partum and postpartum periods to achieve successful estrus and breeding performance [8]. Particular nutrients and feeding components affect the fertility in cows, and the nutritional management around the delivery of a calf can influence successful reproduction [3,9].

Human errors about practical knowledge of estrus lead to inadequate estrus expression in high-yielding dairy cows and unfavorable reactions to heat stress result in improper detection of estrus in dairy herds [10]. Thus, the inappropriate timing of insemination due to poor estrus expression and inadequate estrus detection is a frequent experience in most crossbred dairy cows in Bangladesh. The Artificial insemination (AI) breeding system is a dependable technique for herd owners to enhance genetic improvement and control genital problems in their herds. Despite the benefits of AI, a huge proportion of dairy owners practices natural insemination (NI) for their breeding strategy [10,11]. Dairy farmers use the NI breeding system to avoid improper timing of insemination due to poor estrus detection. Moreover, the semen quality in the AI breeding technique deteriorates during transportation from the semen laboratory to the field institutions, which are either due to mismanagement in transportation and distribution of straws from semen laboratory or during handling and storage of semen in field institutions [12,13]. Thus, the AI breeding system was suggested as a typical cause of low conception in dairy herds [13].

Therefore, the experiment was designed to establish suitable management strategies like improving feeding and breeding policy for dairy herds to increase fertility in the repeat breeding (RB) dairy cows. Different feeding strategies like traditional and STD feeding and other breeding systems, including AI and NI breeding systems, were implemented in the RB cows to evaluate fertility improvement.

## Materials and Methods

### Ethical approval

Initially, this experiment was certified by Animal Welfare and Ethical Committee (AWEC), Bangladesh Agricultural University (BAU), Mymensingh-2202, Bangladesh (No. 05/AWEC).

### Study period and selection of study areas

The research work was conducted from January 2016 to December 2019 throughout the different study areas in Bangladesh. The desired populations of dairy cows for the research were selected from the dairy zones of Bangladesh. To achieve the best possible target of the research, there are about five clusters of study areas, such as (i) Gazaria (23°32.5′N 90°36.5′E), Munshiganj, (ii) Sreenagar (23°32.2′N 90°17.5′E), Munshiganj, (iii) Shahjadpur (24°10.2′N 89°35.3′E), Sirajgonj and Rural Development Academy, Sherpur (24°40′N 89°25′E), Bogura, (iv) Karnaphuli (22°13′N 91°48′E) and Patiya (22°30′N 91°98′E), Chattogram, (v) Mymensingh Sadar (24°45′N 90°25′E), including BAU Veterinary Teaching Hospital, Mymensingh; Gazipur Sadar (24°0′N 90°25.5′E), Gazipur and Savar Military Farm, Savar (23°51′30″N 90°16′00″E), Dhaka.

### Selection of experimental RB cows

A total of 80 RB cows with cyclic ovaries, poor body conditions, low intensity of estrus, and clear vaginal mucus at estrus were randomly selected from the surveyed cows throughout the study areas in Bangladesh. The cows were selected as RB cows in the study, which failed to get pregnant by three or more breeding with regular intervals without notable genital problems as described by Gustafsson and Emanuelson [14].

### Experimental design

Routine deworming in RB cows was carried out using broad-spectrum anthelmintic Trilev-Vet^®^ Bolus. This anthelmintic was administered in the RB cows at the rate of two boluses per 100 kg body weight, and the second dose was applied at 7-day intervals. After 15 days of deworming, RB cows were randomly assigned into four groups (TF1, TF2, SF1, and SF2), and each group comprised 20 cows. RB cows of Group–TF1, Group–TF2, Group–SF1, and Group–SF2 were allocated for traditional feeding practice and NI, traditional feeding practice and AI, STD feeding practice and NI, and STD feeding practice and AI, respectively. 

### Preparation of STD feeds and feeding strategy

The STD feeds were prepared in this experiment, as described by Banerjee [15], to avoid the nutritional imbalance. The detailed chart of this STD feeds and STD feeding strategy for the experimental RB cows are presented in Tables 1 and 2, respectively.

### Selection and management of fertile bulls

The Holstein Friesian crossbred (HF X) (approximately 75%) bulls for the NI breeding system in this experiment were selected from the local areas of study. The bulls were allocated with the history of proven fertility in earlier services and free from venereal diseases. The detailed procedure for the selection and management practices of bulls for NS was followed, as that reported in Risco *et al.*;s [10] study.

### Implementation of feeding and breeding strategies

The RB cows of Group–TF1 (*n* = 20) and Group–TF2 (*n* = 20) were fed by traditional feeding practice and inseminated by NI and AI, respectively, at the proper time. Simultaneously, the RB cows of Group–SF1 (*n* = 20) and Group–SF2 (*n* = 20) were fed by STD feeds and feeding strategies (as shown in Tables 1 and 2) for 90 days. Then, the cows of Group–SF1 and Group–SF2 were inseminated by NI and AI, respectively, at the proper time in the subsequent estrus. NI was carried out by selected HF X (approximately 75%) bulls, and AI was carried out by 0.5 ml frozen semen of HF X (75%) bulls with maintaining proper time and technique.

### Measurement of the dominant ovulatory follicle

The diameter of the dominant follicle (DF) in the experimental RB cows was measured at the time of insemination through ultrasonography using a portable ultrasound scanner (Bionet^®^ MU1V, Korea) assembled with a real-time B mode 7.5 MHz linear transrectal probe. The transrectal ultrasonography was carried out as described by Sharma *et al.* [16]. Ultrasonographically, the DF is a circular, echogenic compact shape, with a highly demarcated border, exhibiting the exterior hyperechogenic layer surrounding the antrum of follicles, representing the follicular wall [17]. The diameter of each and every DF in the cows was measured by intrinsic measuring calipers of an ultrasound scanner thrice, and the mean values (mm) were noted.

**Table 1.** STD feeds for experimental cows.

Feed ingredients		Quantity
Roughages	Dry roughages like hay and rice straw	As per body requirements
	Matured legumes and non-legume’s green grass	
Concentrate mixture	Corn (*Zea mays*)	25%
	Wheat (*Triticum aestivum*) bran	20%
	Rice (*Oryza sativa*) polish	15%
	Khesari dal (*Lathyrus sativus*)	20%
	Soya bean (*Glycine max*) meal	17%
	Di-calcium phosphate	2%
	Sodium chloride salt (NaCl)	1%
	Total	100%

**Table 2.** STD feeding strategy for experimental cows.

Feeds	Quantity	
Roughage and concentrate mixture as total dry matter @ 2.5 kg per 100 kg body weight	Roughages–66%	Dry roughages–66%
		Green roughages–34%
	Concentrate mixture–34% (as per Table 1)	
In milking cow supplementation of 1 kg concentrate mixture for every 3 l of milk production.		
All the feed ingredients should be separated into two portions and supplied twice daily in the morning and evening after milking.		
*Ad libitum* clean drinking water.		

### Estimation of erythrocytes indices

Four ml of blood sample was compiled from experimental RB cows during breeding by the sterile heparin (anticoagulant)-coated vacuum tube and blood collecting needle (VIP^TM^, Zhejiang Gongdong Medical Technology Co., Ltd., South Gate, China) for erythrocytes’ indices. The erythrocytes’ indices like total erythrocyte count (TEC), density of hemoglobin (Hb), and packed cell volume (PCV) were estimated from the collected blood samples within 1 h after sampling. The detailed procedure was completed as described by Lamberg and *Rothstein* [18]. The procedure was carried out thrice for each sample and then the average values were recorded for further analysis.

### Quantitative determination of serum biochemicals and estrogen (E_2_) hormone

Four ml of blood sample was compiled from experimental RB cows during breeding by the sterile clot activator vacuum tube and blood collecting needle for the quantitative determination of serum biochemicals and E_2 _hormone. The blood sample containing tubes was placed at a normal temperature at 45° angles for serum separation. After 4–5 h, the uppermost light yellowish liquid (serum) of the tubes were collected into clean and dry sterile centrifuge tubes with the help of an individual sterile dropper. Then, the serum was centrifuged at 3,000 rpm for 15 min using a centrifuge machine then the upper clearest liquid was collected into another centrifuge tubes. This process was repeated thrice, and the pure serum samples were collected in the properly labeled Eppendorf tube. These Eppendorf tubes containing serum samples were transferred to the laboratory by maintaining a cool chain using a Thermo flask containing ice cubes and stored in the deep freezer at −20°C.

Quantitative determination of biochemical elements like total cholesterol (TC), total protein (TP), glucose, calcium (Ca), phosphorus (P), ferric iron (Fe), copper (Cu), zinc (Zn), and magnesium (Mg) were completed from the preserved serum samples. It was conducted using specific test kits and ultraviolet-visible spectrometer (T80 UV/VIS Spectrometer, PG Instruments Limited, Lutterworth, UK) at Professor Mohammad Hossain Central Laboratory, BAU, Mymensingh-2202, Bangladesh. Detailed procedure was carried out as per the prescribed literature supplied with specific biochemical element test kit. The procedure was carried out thrice for each sample then the average value was recorded for further analysis.

**Figure 1.**
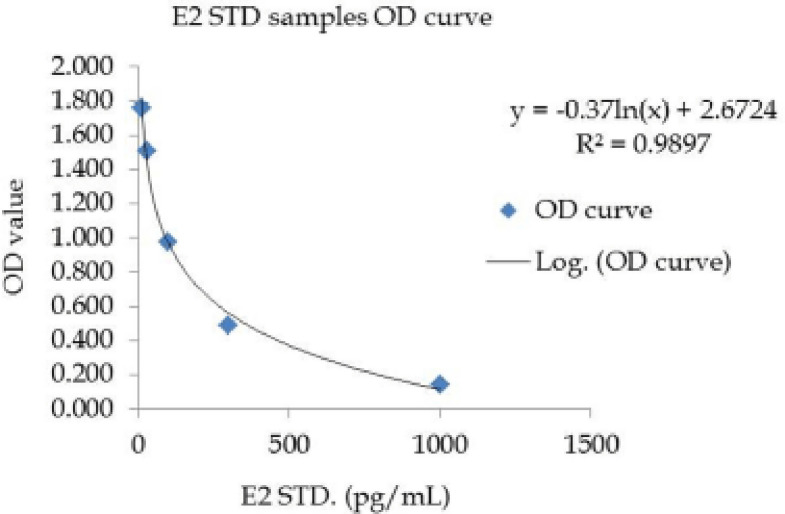
E_2 _STD samples OD curve.

The serum E_2_ hormone was also estimated from the preserved serum samples using a specific hormone enzyme linked immunosorbent assay kit (NovaTec Immunodiagnogtica GmbH, Technologio and Waldpark, Dietzenbach, Germany) and microplate reader (SPECTRAmax^®^ 340PC384, Sunnyvale, CA) in the laboratory of Community Based Dairy Veterinary Foundation, BAU, Mymensingh-2202, Bangladesh. A detailed procedure for estimating optical density (OD) value as ‘*x*’ of E_2 _hormone was carried out as per prescribed literature supplied with the specific test kit. The procedure was repeated thrice for each sample, and the average OD value was recorded for the calculation of serum E_2 _concentration. Concentrations were estimated by the use of formula, *y* = −0.37ln(*x*) + 2.672 for E_2_ calculation which is obtained from the STD samples OD curves (Fig. 1).

### Monitoring of non-return rate and pregnancy diagnosis

**Figure 2.**
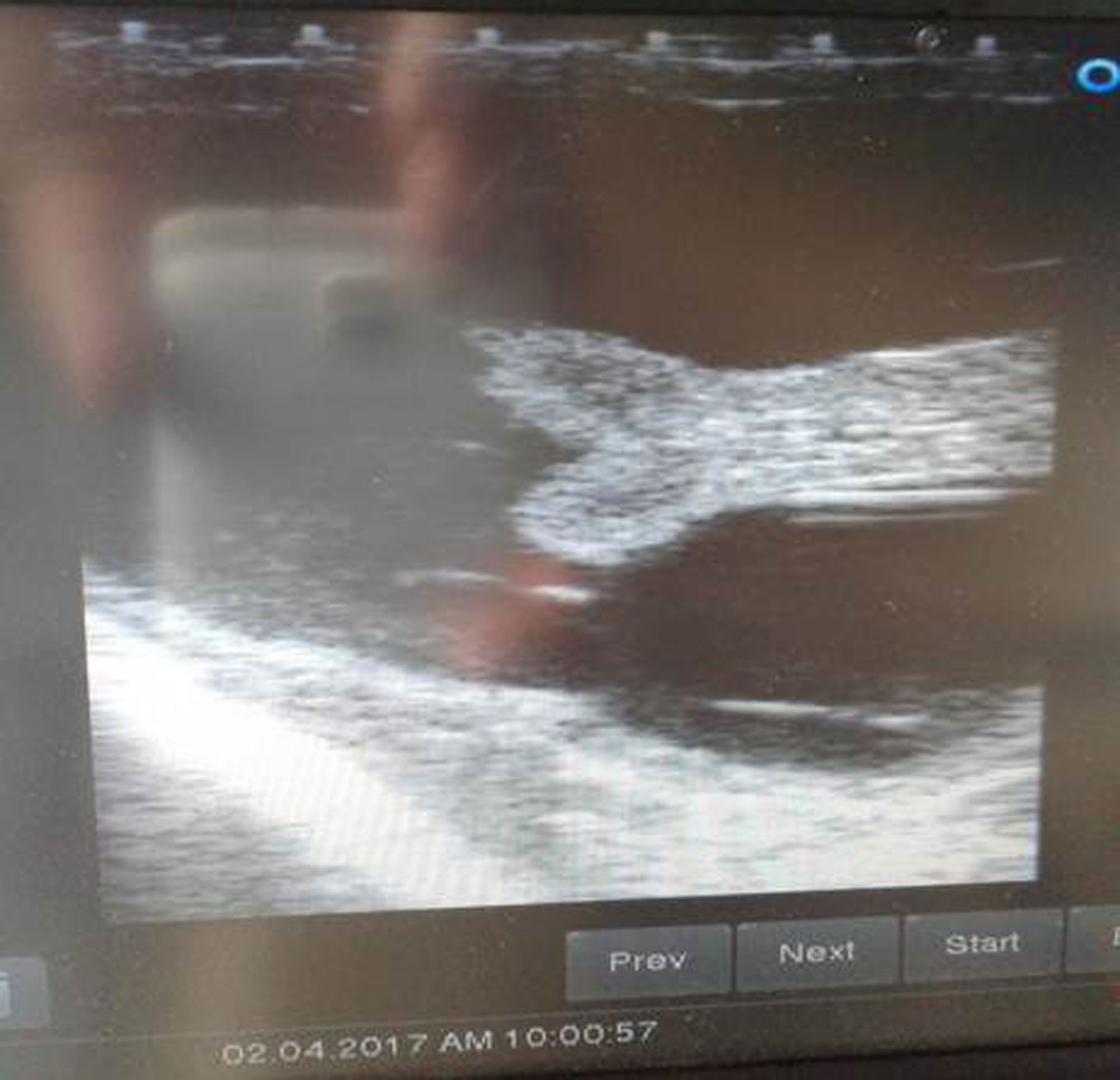
Gravid uterus with 48-day-old live fetus in experimental RB cows.

In inseminated RB cows, the non-return rate was recorded at day 21 post-insemination. Pregnancy diagnosis was also carried out by rectal palpation and transrectal ultrasonography from day 45 to 90 post-insemination to enumerate the effects of different feeding and breeding strategies on fertility in RB cows. The transrectal ultrasonography was carried out as described in an earlier section. Figure 2 shows the gravid uterus with 48-day-old live fetus in the experimental RB cows detected by transrectal ultrasonography.

### Statistical analysis

The chi-square test was carried out to calculate the significant variation in pregnancy rate in RB cows through the effects of different managemental strategies with a 95% confidence interval. Independent samples *t*-test was also carried out to compare the mean value of DF diameter, erythrocytes indices, and serum biochemicals during insemination between traditional and STD feeding groups of RB cows to evaluate the factors affecting in pregnancy rate. Bivariate Pearson’s correlation coefficient was also carried out to correlate among DF diameter, erythrocytes indices, and serum biochemicals at the time of insemination. The statistical approaches in this study were made by using computerized International Business Machines Corporation Statistical package for the Social Science statistics software version 20 (UK).

**Figure 3.**
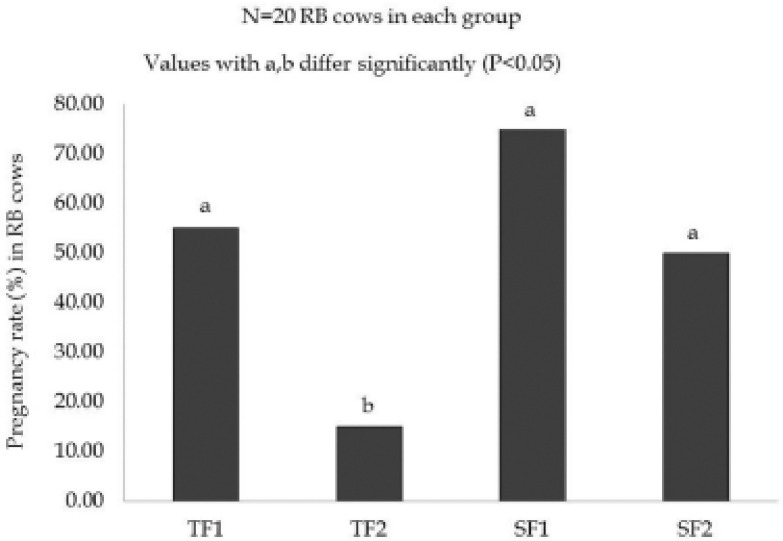
Effect of STD feeds and breeding strategies on the proportion of pregnancy in experimental RB cows.

## Results

The effect of STD feeds and breeding strategies on the proportion of pregnancy in RB cows is shown in Figure . The pregnancy rate in Group–TF1, Group–TF2, Group–SF1, and Group–SF2 were 55.00%, 15.00%, 75.00%, and 50.00%, respectively. The pregnancy rate was higher in Group–SF1 in comparison to Group–TF2 with significance (*p *< 0.05), and it was also non-significantly (*p *> 0.05) elevated in Group–SF1 than Group–TF1 and Group–SF2.

**Figure 4.**
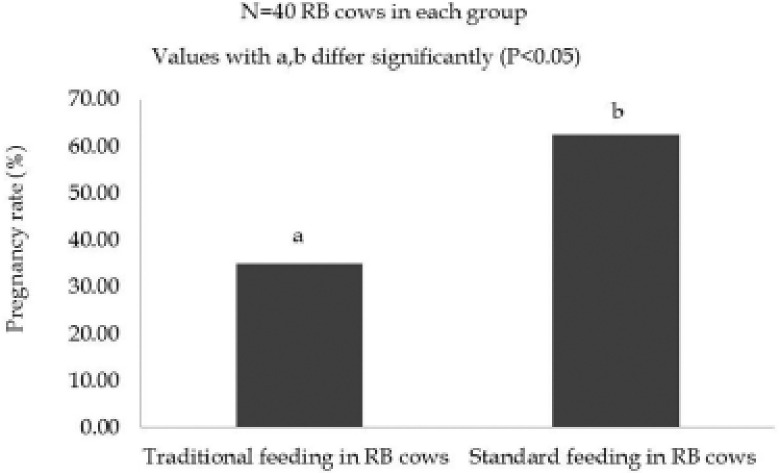
Effect of feeding strategies on the proportion of pregnancy in experimental RB cows irrespective of breeding strategies.

**Figure 5.**
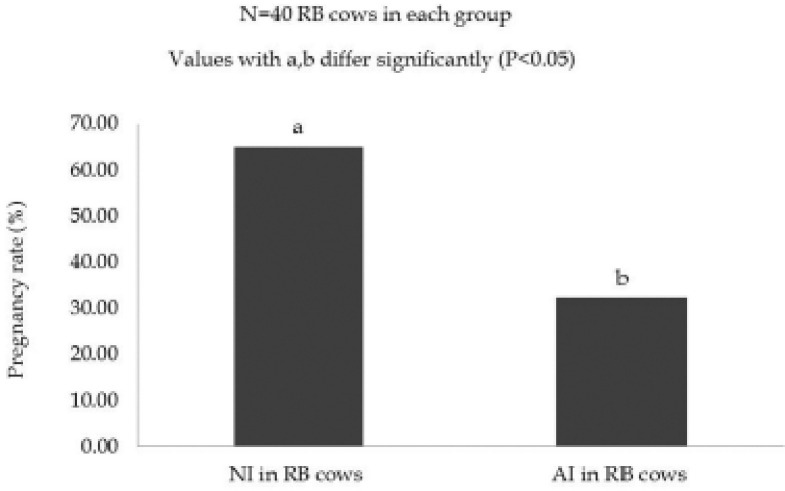
Effect of breeding strategies on the proportion of pregnancy in experimental RB cows irrespective of feeding strategies.

The effect of feeding strategies on the proportion of pregnancy in RB cows irrespective of breeding strategies is shown in Figure 4. The pregnancy rate in traditional feeding practice and STD feeding practice was 35.00% and 62.50%, respectively. The pregnancy rate was higher with significance (*p *< 0.05) in STD feeding practice than the traditional feeding practice in the RB cows irrespective of breeding strategies.

The effect of breeding strategies on the proportion of pregnancy in RB cows irrespective of feeding strategies is shown in Figure 5. The pregnancy rate in NI and AI breeding systems was 65.00% and 32.5%, respectively. The pregnancy rate was significantly (*p *< 0.05) increases in NI compared to the AI breeding system in the RB cows irrespective of feeding strategies.

Effect of feeding strategies on ovarian and hemato-biochemical parameters during breeding in the experimental RB cows irrespective of breeding strategies is shown in Table 3. The diameter of DF, serum E_2_, TEC, Hb, PCV, TC, TP, glucose, Ca, P, Fe, Cu, Zn, and Mg at the time of insemination were greater with significant (*p *< 0.01) in the experimental RB cows with STD feeding practice compared to traditional feeding practice.

Pearson’s correlation among DF diameter, serum E_2,_ and hemato-biochemicals in the experimental cows at the time of insemination is shown in Table 4. The diameter of DF was significant (*p *< 0.01) positively correlated with serum E_2_, TEC, Hb, PCV, TC, TP, glucose, Ca, P, Fe, Cu, Zn, and Mg. Simultaneously, the serum E_2_ was also significant (*p *< 0.01) positively correlated with TEC, Hb, PCV, TC, TP, glucose, Ca, P, Fe, Cu, Zn, and Mg at the time of insemination in the experimental RB cows.

**Table 3.** Effect of feeding strategies on ovarian and hemato-biochemical parameters at insemination in experimental RB cows irrespective of breeding strategies.

Parameters	STD feeding in RB cows	Traditional feeding in RB cows
Diameter of DF (mm)	14.11 ± 0.29^**^	9.10 ± 0.25
Serum E_2_ (pg/ml)	15.18 ± 0.28^**^	8.33 ± 0.17
TEC (million/μl)	9.48 ± 0.14^**^	7.82 ± 0.10
Hb (g/dl)	9.69 ± 0.12^**^	6.69 ± 0.08
PCV (%)	37.88 ± 0.33^**^	28.44 ± 0.48
TC (mg/dl)	208.81 ± 1.84^**^	165.68 ± 3.87
TP (g/dl)	9.48 ± 0.29^**^	5.36 ± 0.34
Glucose (mg/dl)	74.75 ± 1.40^**^	43.74 ± 4.27
Ca (mg/dl)	8.63 ± 0.27^**^	5.89 ± 0.18
P (mg/dl)	5.96 ± 0.11^**^	2.11 ± 0.08
Fe (μg/dl)	365.00 ± 1.66^**^	340.28 ± 2.17
Cu (μg/dl)	74.07 ± 1.18^**^	61.48 ± 1.07
Zn (μg/dl)	174.00 ± 1.90^**^	119.92 ± 2.83
Mg (mg/dl)	2.62 ± 0.07^**^	1.72 ± 0.02

*n* = 40; RB cows in each experimental group. Values are Mean ± SE.

** Significant at *p *< 0.01 within the row.

## Discussion

In this experiment, the selected RB cows with clear vaginal mucus at estrus, cyclic ovaries, poor body grade, and poor nutritional status were considered to establish suitable management strategies by executing different feeding strategies and different breeding systems. The traditional and STD feeding strategies were practiced for 90 days, and then the RB cows were bred through AI and NI breeding system in both feeding strategies.

Results illustrated that the pregnancy rate was increased in STD feeding practice with NI. The pregnancy rate was also elevated in STD feeding practice than traditional feeding practice in the experimental RB cows. The STD feeding strategies were practiced in this experiment described by Banerjee [15], enriched with essential nutrients, vitamins, and minerals. In the present study, this STD feeding strategy was practiced in the selected RB cows for 90 days before insemination compared to the traditional feeding practice by farmers. As a result, the ovarian profiles (DF diameter and serum E_2_) and hemato-biochemical parameters in the body were adjusted to physiological level (as reported by de Tarso *et al.* [19], Jackson and Cockcroft [20], Naik *et al.* [21]) in the treated RB cows with STD feeding practice. In this consequent, the result exposed that the DF diameter and serum concentrations of E_2_, TEC, Hb, PCV, TC, TP, glucose, Ca, P, Fe, Cu, Zn, and Mg at insemination were elevated in STD feeding practice compared to traditional feeding practice in the experimental RB cows at the time of insemination. In the present findings, the Pearson correlation showed that the diameter of DF was positively correlated with serum E_2_ concentration, and the E_2_ was positively correlated with serum TC at estrus, which is similar to the earlier reports [22-25]. During insemination, the greater DF has been linked with increased circulating E_2_ and results in higher estrus assertion intensity [24,25]. The pregnancy and estrus assertion are positively correlated, which is undoubtedly related to higher circulating E_2_ in females [23,24]. The DF diameter and E_2 _were also positively correlated with the serum biochemicals in this experiment. The increasing level of hemato-biochemical elements like TEC, Hb, PCV, TC, TP, glucose, Ca, P, Fe, Cu, Zn, and Mg improve the pregnancy rate in animals in many ways [26-35]. Consequently, the fertility in the experimental RB cows with STD feeding strategies was improved in this experiment. 

**Table 4.** Pearson’s correlation among DF diameter, serum E_2_, and hemato-biochemicals in experimental cows at the time of insemination.

Indices	Correlation (*r*)*p*-value	E_2_	TEC	Hb	PCV	TC	TP	Glucose	Ca	P	Fe	Cu	Zn	Mg
DF	*r*	0.863^**^	0.577^**^	0.754^**^	0.678^**^	0.640^**^	0.586^**^	0.491^**^	0.596^**^	0.756^**^	0.682^**^	0.549^**^	0.718^**^	0.674^**^
	*p*-value	0.000	0.000	0.000	0.000	0.000	0.000	0.000	0.000	0.000	0.000	0.000	0.000	0.000
E_2_	*r*		0.649^**^	0.823^**^	0.789^**^	0.702^**^	0.661^**^	0.581^**^	0.705^**^	0.861^**^	0.718^**^	0.644^**^	0.772^**^	0.717^**^
	*p*-value		0.000	0.000	0.000	0.000	0.000	0.000	0.000	0.000	0.000	0.000	0.000	0.000

** Significant at *p *< 0.01.

Many scholars in recent years [3,4,9,36,37] reported that balanced nutrition influences the fertility in cattle, which is similar to the present findings. The feasible relation between nutrition and reproduction in the cows is reported, and various nutrients differ markedly . Moreover, the cows are normally fed with huge balanced nutrients; thus, nutritional scarcity is very unlikely to occur. Upgraded and proper feeding enhanced the performance of reproduction and improved the reproductive traits [4,7,38]. Production of circulating hormones in the cows modulated by energetic condition, which plays the critical roles in maturing follicles, ovulation, corpus luteum formation, and oocyte capability. Simultaneously, excessive lipolysis and fat metabolism are associated with a deleterious effect on the capability of ovulatory follicles and the growth of conceptus. But the fertility proportion and embryo quality in the cows are affected by certain fatty acids [3]. Rodney *et al.* [9] also reported that the dietary fat, energy, and protein symmetries in early lactation are very important for elevated pregnancy results and finally propose that the starch and sugars might have various effects on pregnancy rate inseminations.

The result also exposed that the pregnancy rate was also improved in the NI breeding system than the AI breeding system. Similar to the present study, many authors also opined that the pregnancy rate was higher through the NI breeding system in the dairy cows than AI [10,39,40]. The NI breeding system increases the chance of conception rate due to the timing of semen deposition is good in the NI breeding system than that of AI [40]. Furthermore, in the herds with poor estrus detection, NI results in a higher pregnancy due to human faults in heat detection are avoided when the use of bulls in the NI breeding system [10,39]. Simultaneously, the NI breeding system might influence the pregnancy rate in cows due to harmful effects of *in-vitro* storage of spermatozoa, deteriorated spermatozoon quality during transportation, improper handling of frozen semen, indecent site of deposition in the female genital tract, and poor skills of inseminators are also avoided during the bulls are used in NI than AI breeding system. Thus, the NI breeding system over the AI increases the fertility in the experimental RB cows.

## Conclusion

The present experiment indicated that the supply of STD feeds and feeding strategies could increase the ovarian, hormonal, and hemato-biochemical status in the experimental RB cows. These STD feeds and feeding strategies with the NI breeding system improve the fertility in the RB cows.

## List of Abbreviations

AI, Artificial insemination; AWEC, Animal Welfare and Ethical Committee;, ;BAU, Bangladesh Agricultural University; *, *;Ca, calcium;, ;Co., company; Cu, copper; DF, dominant follicle; E_2_, estrogen;, ;et al., associates; Fe, ferric iron; Hb, hemoglobin; HF X, Holstein Friesian crossbred;, ;kg, kilogram;, ;Ltd, limited; Mg, magnesium; MHz, megahertz; mL, milliliter; mm, millimeter;, ;NI, natural insemination; NS, natural service; OD, optical density; P, phosphorus; PCV, packed cell volume; RB, repeat breeding;, ;, ;rpm, rotation per minute;, ;STD, standard; TC, total cholesterol; TEC, total erythrocyte count; TP, total protein; Zn, zinc; %, percent; <, lesser than; >, greater than; ^®^, registered trademark; °C, degree celsius.
